# *Drosophila melanogaster* diabetes models and its usage in the research of anti-diabetes management with traditional Chinese medicines

**DOI:** 10.3389/fmed.2022.953490

**Published:** 2022-08-11

**Authors:** Yaodong Miao, Rui Chen, Xiaolu Wang, Jie Zhang, Weina Tang, Zeyu Zhang, Yaoyuan Liu, Qiang Xu

**Affiliations:** ^1^Second Affiliated Hospital of Tianjin University of Traditional Chinese Medicine, Tianjin, China; ^2^School of Traditional Chinese Medicine, Tianjin University of Traditional Chinese Medicine, Tianjin, China; ^3^Jimo District Qingdao Hospital of Traditional Chinese Medicine, Qingdao, China; ^4^School of Pharmaceutical Science and Technology, Tianjin University, Tianjin, China; ^5^First Teaching Hospital of Tianjin University of Traditional Chinese Medicine, National Clinical Research Center for Chinese Medicine Acupuncture and Moxibustion, Tianjin, China

**Keywords:** *Drosophila*, diabetes, insulin pathway, TOR pathway, AKH pathway, TCM

## Abstract

The prevalence of diabetes mellitus (DM) is increasing rapidly worldwide, but the underlying molecular mechanisms of disease development have not been elucidated, and the current popular anti-diabetic approaches still have non-negligible limitations. In the last decades, several different DM models were established on the classic model animal, the fruit fly (*Drosophila melanogaster*), which provided a convenient way to study the mechanisms underlying diabetes and to discover and evaluate new anti-diabetic compounds. In this article, we introduce the *Drosophila* Diabetes model from three aspects, including signal pathways, established methods, and pharmacodynamic evaluations. As a highlight, the progress in the treatments and experimental studies of diabetes with Traditional Chinese Medicine (TCM) based on the *Drosophila* Diabetes model is reviewed. We believe that the values of TCMs are underrated in DM management, and the *Drosophila* Diabetes models can provide a much more efficient tool to explore its values of it.

## Background

In the last decades, changes in lifestyles have led to a dramatic increase in the prevalence of diabetes mellitus (DM). In 2017, there were approximately 425 million diabetics all over the world, and it is estimated that by 2045, the number of diabetics will reach 629 million (1, 2). An epidemiological study in 2018 showed that about 11% of people in China suffer from DM (3), and DM has then become one of the most important healthcare challenges. The patients suffering from DM have symptoms including weight loss, frequent urination, tired feeling, and sores that cannot be healed. Furthermore, hyperglycemia can induce multi-system complications, especially the chronic damage and dysfunction of the eyes, kidneys, heart, nerves, and blood vessels, which seriously reduce the patients’ quality of life. Although various diabetic drugs are currently available, many of which have hypoglycemic action, there are still some limitations that exist. It is difficult to precisely control the blood glucose level, while the drug resistance and it’s intolerant and side effect on different individuals are also a huge problem. A more personalized DM management strategy considering patients’ comorbid conditions and social context requires a better understanding of the exact basis of the DM mechanism and further exploration of anti-diabetic tools in our arsenal (4, 5).

Many animal models have been established for DM studies, including rabbits, rats, and mice, but few people have used invertebrates to systematically study DM and related metabolic diseases (6). In the late 1970s, insulin-like peptides (ILPs) were first isolated in the brain of fruit flies, which developed a new animal model that could be used for DM study (6). In the last decades, several *Drosophila* Diabetes models were established and used to elucidate the molecular mechanism underlying the disease development of DM and to evaluate the prospective drug compounds against DM or its induced complications. In 2002, the first *Drosophila* Diabetes model was established, and the removal of insulin-producing cells (IPCs) in the fruit fly brain could produce phenotypes that were similar to that of human type 1 diabetes, including elevated blood glucose level, weight loss, and developmental delay. These studies reveal that the IPCs in the fruit fly brain are a functional ortholog of human Islet β cells, and the fruit fly shares a common cellular mechanism in blood glucose regulation with humans (7), which builds the fundamentals of using fruit fly to study human diabetes.

The fruit fly has obvious advantages as a diabetes model: (1) it is small and can be easily raised in large quantities in the laboratory (8); (2) it has a short life-cycle (10–12 days) and thus it is the data results of certain studies based on it that can be obtained quickly; (3) it has complex neuropils and neural circuits and rather complicated behaviors; (4) it only has four chromosomes in relatively smaller genome size, but those can match 75% pathogenic genes of human (9); (5) its genome is well-annotated in complete sequence; (6) dozens of powerful genetic tools are developed on fruit fly, which can provide convenient approaches to achieve various genetic manipulations, including UAS-GAL4, LexA-lexAop, QF-QUAS, and mosaic analysis; (7) The reduction of the cost of experimental research. The mechanism of glucose homeostasis regulation in fruit flies is highly conserved with that of humans in a genetic context (10). When a fruit fly feeds, the sugars in the food are transported through the digestive and intestinal tracts into its gut epithelial cells and are converted into trehalose as the major saccharide for the insect circulatory system. Then the trehalose is transported into the hemolymph which is the “blood” of insects, and the excess sugar is stored in the form of glycogen or triglyceride (TAG) in fat bodies and muscles. The function of the fruit fly fat body is similar to that of the liver and adipose tissue in mammals except for glycogen stores, and it is responsible for regulating the body’s energy metabolism (11, 12). The IPCs of fruit fly are similar in function to human islet β-cells as well, producing *Drosophila* insulin-like peptides (DILPs) to lower blood glucose (13), and also the presence of the adipokinetic hormone (AKH), an analog of glucagon, undertakes the upregulation of blood glucose level. It is the two hormones (DILPs and AKH) that antagonize to regulate the blood glucose homeostasis in fruit flies (14).

Traditional Chinese medicine (TCM) is a treatment system for thousands of years. It has long been used in China to treat diabetes and its complications. Many traditional medicinal plants, natural products, and complicated prescriptions are still used in the clinical practices of Chinese doctors every day and are considered effective. It is a potential treatment for diabetes control. However, the lack of scientific or medical scrutiny and molecular mechanism research strongly inhibit the development and usage of such effective traditional medicines. We suggest using the most convenient and convinced *Drosophila* Diabetes model to investigate such traditional Chinese medicines, screen out bioactive compounds, and study the underlying molecular mechanisms of such natural products to treat Diabetes, which will contribute to the methods of treating diabetes and related complications (15, 16).

In this paper, we introduce the signal pathways involved in glucose homeostasis regulation and energy metabolism, which exist in both fruit flies and humans, describe the methods to establish *Drosophila* Diabetes models, and use them in pharmacodynamic evaluations. At last, we also summarize the research improvement which analyzes how traditional Chinese medicine works in managing Diabetes based on *Drosophila* Diabetes models.

## Signal pathways

**FIGURE 1:**
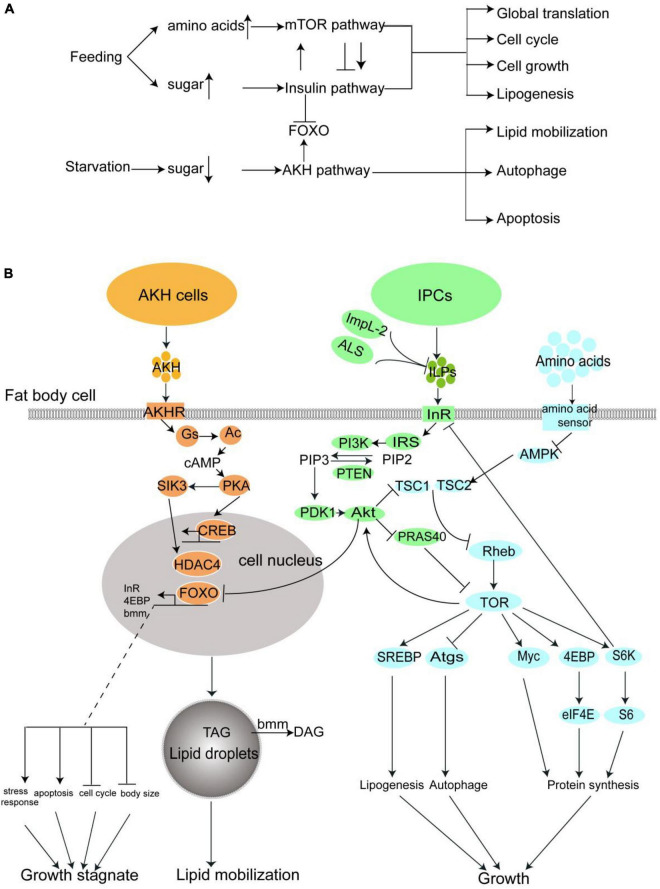
The nutrient metabolism of fruit fly are regulated major by three pathway: The insulin pathway, the mTOR pathway, and the AKH pathway. **(A)** A summary of the interaction and effect of these three pathways. **(B)** A more detailed diagrammatic sketch of these three pathways.

Same to mammals, the regulations of energy metabolism in insects are achieved mainly *via* insulin/IGF signaling (IIS), TOR signaling and AKH signaling (Figure 1). These three pathways not only regulate the carbohydrate storage and mobilization according to nutrient conditions, but also mediate protein synthesis, cell survival, and proliferation, and control the development and body growth of insects together with Ras/MAPK signaling, JNK signaling, and ecdysone signaling (17, 18). All the pathways, except the ecdysone signaling pathway, are conserved in animals, including insects and humans, which allows us to establish models of diabetes and tumors in the fruit fly to mine the detailed interactions of such pathways and diseases, and further find targeted therapies (19–21).

In this section, we will make a sketch of insulin signaling, TOR signaling, and AKH signaling in the fruit fly, which directly relates to diabetes in both insects and humans. A much more detailed introduction of the interaction of Diabetes related pathways and genes were well-summarized in the review written by Álvarez-Rendón et al. (22).

### Insulin signaling

As mentioned in the previous section, the insect insulin orthologous ILPs are produced and released from insulin produce cells (IPC) into hemolymph when the blood glucose keeps on a high level. The ILPs are received by the Insulin receptor (InR) on the surface of fat body cells. The fat body, with another cell type, the monocytes gathered to form the functional ortholog of adipose tissue and liver, is the center of energy metabolism in the insect body. InR is a ligand-stimulated tyrosine kinase, which induces a phosphorylation cascade when ILPs exist. The phosphorylation cascade sequentially phosphorylates the Insulin receptor substrate (IRS/Chico) and PI3K (17, 23). The PI3K catalyzes the phosphorylation of phosphatidylinositol 4,5-bisphosphate(PIP2) to phosphatidylinositol 3,4,5-bisphosphate (PIP3) in the cell membrane (24). This reaction can be reversed by phosphatase and tensin homolog (PTEN) (25). Enrichment of PIP3 in cell membrane accumulates further phosphoinositide-dependent protein kinase 1 (PDK1) and Akt *via* the binding between their PH domain and PIP3. PDK1 then actives Akt by phosphorylation(p-Akt), and p-Akt further phosphorylates a number of different downstream proteins. The most important one is the Forkhead Box subfamily O transcription factor, or FOXO, which regulates the expression of various downstream genes that are involved in energy metabolism, cell cycle, stress response, and apoptosis (26). The phosphorylated FOXO loses its transcriptional activity (27). It is noteworthy that *InR* itself is also a target gene of FOXO, which is assumed to serve as a negative feedback mechanism of insulin signaling.

### *TOR* signaling

The mechanistic target of the rapamycin (mTOR) pathway is another signaling pathway controlling nutrient usage and cell growth. mTOR is a rapamycin-sensitive tyrosine kinase located at the center of this pathway. The activity of mTOR closely interacts with insulin signaling. p-Akt in insulin signaling activates mTOR *via* inhibition of the tuberous sclerosis complex 2(TSC2) and PRAS40, two inhibitors of TOR activity (28–33). TOR signaling activation can also be directly regulated by dietary amino acids (34). The uptake of amino acids in hemolymph is conducted by transporters, such as Slif, Mnd, and Path into the cells. High amino acids level in the cell suppresses another mTOR suppressor AMPK (35), and thus promote the mTOR activity (36). The activation of mTOR stimulates global protein synthesis through phosphorylation of the translation initiation factor 4EBP, another transcription factor Myc, and ribosomal protein S6 kinase. mTOR also promotes lipogenesis and represses autophagy *via* phosphorylation of transcription factor SREBP (37, 38) and autophagy proteins (39), respectively. In general, the activation of mTOR leads to cell growth and general biosynthesis while suppressing catabolism (40).

The TOR signaling also mediates the activity of the insulin pathway: mTOR directly enhances the Akt activation (41), while S6K is negative feedback, which downregulates the IRS activity (42).

### Adipokinetic hormone signaling

The adipokinetic hormone is the insect analog of human glucagon (43). Similar to ILPs, AKH is produced by a special cell set, called Corpora Cardiac Cells (CCCs) in the neuroendocrine ring glands (44). The CCCs Cells, which recept the low trehalose level in the hemolymph by energy sensor on their surface, activate the AMPK signaling and subsequently induce the opening of the Ca^2+^ channel to elevate the Ca^2+^ concentration in the cells, which results in the release of AKH to the hemolymph (45).

Adipokinetic hormone is received by AKHR which also mainly presents on the cell surface of the fat body. The AKHR is a G-protein coupled receptor, which activates G-protein after it binds with AKH, the first messenger here, leading to the dissociation of Gs small G-protein subunit from the G-protein, and activation of adenylate cyclase (Ac). The Ac produces cAMP from ATP as the second messenger, which further actives protein kinase A (PKA). PKA further transduces the signal *via* phosphorylates transcription factor CREB and salt-induced kinase 3 (SIK3). The SIK3 controls the activity of histone deacetylase 4(HDAC4) and further mediates the activity of FOXO. In one sentence, the AKH activates two transcription factors, FOXO and CREB, which lead to lipid mobilization, and cell growth stagnation in the fat body to face starvation (46). For example, one well-known FOXO target gene Brummer lipase gene (*bmm*) is upregulated by AKH signaling, which catalyzes the conversion of TAG to diglyceride (DAG) and releases free fatty acids from fat body lipid droplets.

In summary, feeding activates the insulin signaling and TOR signaling, which lead to global biosynthesis and cell growth *via* inhibition of FOXO, while starvation activates the AKH signaling, which in turn activates the FOXO, promotes nutrient mobilization, and blocks the growth. In Figure 1, we drew these three pathways in one diagrammatic sketch to feature the interaction between them.

## Establishment of *Drosophila* Diabetes models

**FIGURE 2:**
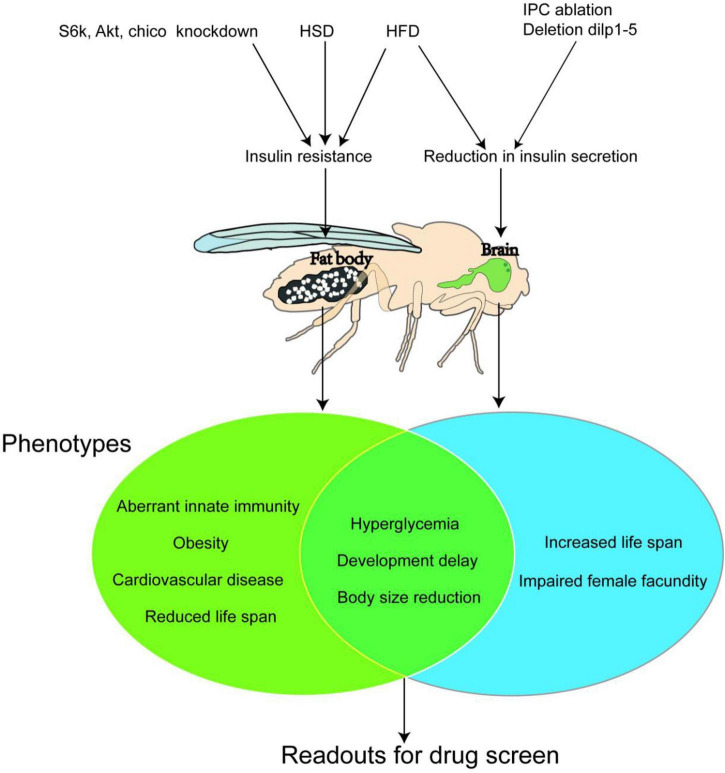
Three classic *Drosophila* Diabetes Models. A high sugar diet (HSD) and High-fat diet (HFD) affect the nutrient metabolism in both larvae and adult bodies (especially the fat body) of fruit flies and induce insulin resistance, which causes type 2 diabetes. While ablation of the insulin-produced cells (I) or knockdown of the expression of *insulin-like peptides* (*dlips*) in their brain abolish the secretion of DLIPs, thus building a type 1 diabetes model. The Venn diagram presents the symptoms (or phenotypes) of the type 2 diabetes model and type 1 diabetes models of the fruit fly. Both models show common marks, such as hyperglycemia, developmental delaying, and growth retardation. Such marks could be used as trails for pharmacodynamics evaluation during large-scale new anti-diabetic compounds screening.

The *Drosophila* Diabetes model is now widely applied to the research of underlying mechanisms on the onset of diabetes and its complications, significantly promoting the discovery rate of new targets and therapeutics with limited cost. Since a variety of DM models has been established in terms of fruit fly, the classic *Drosophila* Diabetes models will be introduced below, including partial or complete elimination of IPC (type 1 diabetes), knockout of Dilp1-5 gene (type 1 diabetes), insulin signaling pathway mutation (type 2 diabetes), and high-sugar or high-fat inducing (type 2 diabetes) (Figure 2).

### Establishment of type 1 diabetes models

The distinguishing pathophysiological feature of type 1 diabetes is the acute reduction or absence of insulin secretion due to the loss of pancreatic islet ß-cells (47). The clinical feature, except for hyperglycemia, is mostly characterized by a young age of onset, polydipsia, polyuria, weight loss of unknown cause, non-obese body size, and appearance of autoimmune markers such as Glutamic Acid Decarboxylase Antibody (GADA) and Insular Cellular Antibody (ICA).

Rulifson et al. (7) performed a complete elimination of fruit fly IPCs through gene modification and found fruit fly larvae showed type 1 diabetes phenotypes such as hyperglycemia, weight loss, and developmental delay. Then Broughton et al. (48) discovered that fruit flies with partial elimination of IPCs could also develop the above phenotypes. Similarly, Zhang et al. (49) in 2009 knocked out the expression of Dilp1-5 genes, which caused fruit flies to present with metabolic defects similar to type 1 diabetes in mammals, such as stunted growth, small size, elevated glucose levels, and poor fertility. These modeling methods verify that the saccharides metabolism homeostasis of fruit flies is very similar to that of mammals and can be used to complete the basic theoretical research of diabetes and new drug development. However, these models can be applied only in fundamental research on type 1 diabetes due to their single clear etiological mechanism and, for the same reason, cannot be widely used in clinical applications.

### Establishment of type 2 diabetes models

Different from type 1 diabetes, the core pathophysiological feature of type 2 diabetes is insulin resistance, accompanied by the decline of insulin’s ability to regulate glucose metabolism (47).

In clinical practice, the development of type 2 diabetes in humans is closely related to unhealthy dietary habits. By the same token, a model of type 2 diabetes can also be produced in fruit flies by feeding a diet high in sugar or fat. In 2011, Musselman et al. (50) first found that the high-sugar-diet (HSD) could induce metabolic disorder in fruit fly larvae, which is similar to that of type 2 diabetes patients. The level of Insulin-like peptides (ILPs) in their hemolymph is notably higher than that in the normal-fed animal, but the phosphorylation level of Akt1 remains unchanged. These two indications suggest that the Insulin pathway is not activated by high ILPs level. In other words, it is insulin resistance that occurs and results in a phenotype similar to that of human type 2 diabetes such as hyperglycemia. Moreover, there are further studies showing that HSD-fed fruit fly adults also develop insulin resistance (51, 52) and hyperglycemia. High-fat-diet (HFD) feeding can lead to increased triglyceride (TG) content, causing disorders in insulin and glucose metabolism in fruit flies both adults and larvae. When the level of blood glucose rises under HFD feeding, the ILP2 level in fruit flies increases in the first 2 days. The Akt activity stays lower than the normal range, but the TOR pathway is still activated, presenting as insulin resistance. And then the ILP2 level continues to decline from day 5 to day 10, causing insulin-deficient instead (53).

In addition, both HSD and HFD programs can cause diabetes-related cardiac structural disease. Research on high-fat models has found that inhibiting the TOR pathway can effectively prevent the occurrence of heart disease, indicating that the TOR pathway is a key target for the treatment of cardiac and metabolic diseases (54), and thus the fruit fly models can be used to study the complications of diabetes. The HSD and HFD models realize the establishment of a *Drosophila* Diabetes model by improving the diet. The operation is simple and flexible and does not require specific genetic modification. It greatly stimulates the process of human type 2 diabetes caused by unhealthy dietary habits. The HSD model is the most widely used diabetes model of fruit fly up to date.

Studies Murillo-Maldonado et al. (55), Park et al. (56), and Tatar et al. (57) showed that insulin resistance could be achieved in fruit flies by knocking out and knocking down important elements in the Insulin/TOR pathway. For example, mutants of *InR, chico, Dp110, Akt1, Rheb*, and *S6K* disrupt the Insulin/TOR pathway and cause varying degrees of insulin resistance respectively. These mutants show a series of type 2 diabetes phenotypes, such as developmental delay, small body size, significant lipid increase (obesity), and damage to brain and retina function. The diabetes models with downregulated *S6K*, *Akt1*, and *chico* were complementary to the HSD model in pharmacological research and indicated the effect position of a certainly tested compound on the whole insulin-related pathway. For example, the S6k deficient exhibits an extreme reduction in body size, which is caused by smaller cell size rather than lower cell numbers (58), while Yejuhua (*Chrysanthemum indicum* L.) extract can improve the metabolic disorder caused by HSD. Further survey on the effect of CI extract on *S6k* mutants showed that it could also rescue the cell-autonomous reduction in body size which was caused by the loss of S6k activity. CI extract could also reduce the cell area and the size of droplets, which was upregulated in the fat body of *Akt1* knockdown larvae. These results indicated that CI extract performed its anti-diabetes effect due to interaction with a node of the insulin pathway below *S6k* and *Akt1* (59). Korean red ginseng (KRG) could extend fruit fly lifespan. The longevity effect of KRG disappeared in *chico* heterozygous mutant *chico^l/+^*. In addition, by decreasing the ILPs and increasing insulin antagonist ImpL2 expression levels, the authors concluded that KRG-induced lifespan extension was mediated by direct reduction of ILP signal (60).

In Figure 2, we summarize important *Drosophila* Diabetes models, and their major target organs and phenotypes.

## Disease phenotype and pharmacodynamic evaluation

Artificially induced diabetes in fruit flies can be monitored with several physiological and developmental traits. Some are unified to the disease symptoms in humans, such as hyperglycemia and complicated cardiac diseases, and some are specific to the fruit fly, such as developmental delay. Nevertheless, many of these trials can be used to judge the development level and severity of the disease and evaluate the effectiveness of certain treatments.

### Metabolic functions

In clinical practice, the metabolic function of diabetic patients is mostly assessed by testing glycolipid levels (such as capillary blood glucose, glycated hemoglobin, high-density lipoprotein cholesterol, and triglycerides) and insulin levels (such as insulin release test). Similarly, the measurement of metabolic function in the study of fruit fly diabetes model includes blood glucose level, TAG level, fat accumulation in the fat body, mitochondrial activity level, and DILPs expression level are routinely recorded in diabetic studies (52). The measurement of hemolymph trehalose (blood glucose) level is the core criterion for determining the course of diabetes in fruit flies among others. At present, what is mostly used is the high-speed homogenization method to evaluate the level of sugar metabolism (61, 62) and to determine the trehalose content of the whole body of a fruit fly, which is approximately close to the blood glucose level. There were a few works showing that the hemolymph was extracted with artful methods and the exact blood glucose level was thus determined (63–65) through this method. However, the trehalose level of the whole body is generally enough to evaluate the diabetic level. Lipid metabolism level is often assessed by measuring the content of triglycerides (53) and the accumulation of fat (such as the size of fat body cells, and the area and number of lipid droplets) (13).

### Related indicators of growth and development

Abnormalities in glucose and lipid levels can be preliminarily determined by changes in body weight among typical diabetic symptoms (polydipsia, polyuria, polyphagia, unexplained weight loss), and body mass index (BMI) is also a common indication for observing the efficacy of clinical diabetic patients. Fruit fly diabetic models commonly exhibit developmental delays and growth retardation. In order to explore the alleviating effects of drugs on metabolic abnormalities in diabetic fruit flies, what can be done is to observe (13) its lifespan, individual size, growth rate, body weight, time to pupariation, and other related indices, which are very convenient and intuitive to be measured and thus can be used for large-scale screening of drug candidates. The growth rate can be evaluated by pupariation time and eclosion time (59). The alteration of body size is normally evaluated by pupa size, body weight, eye size, and wing size (58). The alteration of cell size can be assessed by the number of hair on certain wing areas (58) because each wing epidemic cell develops one hair. The measurements of these trails are very convenient and intuitive, and are proper for large-scale screening of new drugs and identifying the active compounds from candidate complexes.

In addition, fertility is inextricably linked to growth and development. By analyzing the egg production rate of female fruit flies and the hatching rate of fertilized eggs, it is found that traditional Chinese medicines (TCMs), such as Jinyinhua (*Lonicera japonica* Thunb.) and Yejuhua (*Chrysanthemum indicum* L.), can improve the fertility and survival of offspring in high-sugar-fed fruit fly (13).

### Changes in behavior

Diabetic neuropathy is one of the most common chronic complications of diabetes, and can often involve both central and peripheral nerves, with the latter being the most common in clinical practice (66). Central neuropathy in diabetic patients has a significant impact on cognitive function, which is mostly assessed in clinical studies based on the simple intelligence checklist (67). Peripheral neuropathy is dominated by distal symmetric polyneuropathy and is often diagnosed based on clinical symptoms, signs, and nerve conduction function tests. The fruit fly brain has complex neuropiles and neural circuits and exists a more complex behavioral capacity. In the study of diabetic flies, flies fed with high-sugar and high-fat diets can also induce rapid aging of the nervous system, which can reflect more comprehensively on the effects of diabetes on the neurological health of fruit flies by observing the corresponding behavioral changes, commonly observed indicators such as motor performance and memory, and studying the diabetic. The molecular mechanism of the formation of concurrent neuropathy thus can be studied. For example, motor performance can be assessed by observing the improvement of drugs on the crawling ability of diabetic fruit flies, and it is possible to screen out TCMs, such as Duzhong (*Eucommia ulmoides* Oliv.) and Yanhusuo (*Corydalis yanhusuo* W.T.Wang), which can alleviate the therapeutic effect of diabetic neuropathy (68).

## Research of traditional Chinese medicines for diabetes mellitus treatment based on *Drosophila* Diabetes models

Traditional medicines, especially traditional Chinese medicines, have a long clinical application history for DM management. Dozens of herbs and natural products from TCM were commonly used and considered effective for DM treatment in China, such as Renshen (*Panax ginseng* C. A. Mey.), Huanglian (*Coptis chinensis* Franch.), Gegen (*Pueraria thomsonii* Benth.), and Zhimu (*Anemarrhena asphodeloides* Bge.) (69–72). In recent years, TCMs have developed rapidly, and the value of traditional Chinese medicines in DM management has gradually become prominent.

Using *Drosophila* Diabetes models to screen the traditional Chinese medicines, which were considered effective in alleviating diabetes, has become a popular research topic in recent years. Here we investigate the published studies about traditional Chinese medicines and their compound prescriptions that apply the *Drosophila* diabetes model to alleviate diabetes and related diseases (Table 1). To pick out major publications, we have searched the database PubMed (using the search term: diabetes; *Drosophila*) and CNKI (using the search term: diabetes; *Drosophila*), and have found 619 papers related to this topic, among which the most important results are summarized below.

**TABLE 1:** Traditional Chinese medicines and their compound prescriptions apply the *Drosophila* Diabetes model to alleviate diabetes and related diseases.

Traditional Chinese medicines	Efficacy
Yejuhua, Duhuo, Juemingzi, Lizhihe, Baizhi, Machixian, Yanhusuo, Zhuling, Baiqian, Yinchen, and Rougui (10)	Alleviating effects on the growth and development of insulin mutant diabetic fruit fly
Duhuo, Juemingzi, and Lizhihe (10)	Improving lipid metabolism disorders and prolong lifespan
Bailian (73)	Relieving insulin resistance induced by high-sugar diet *via* regulating the accumulation of lipids
Pugongying, Zihuadiding, Digupi, Aiye, Zexie, Yinyanghuo, Pipaye, and Juhe (73)	Alleviating effects on insulin resistance induced by the high-sugar diet in fruit fly
Gouqizi (74)	Improving the situation, such as growth retardation and abnormal accumulation of lipid, which are caused by insulin resistance, in a dose-dependent manner
Shihu and Gegen (75)	Synergistic to lower blood glucose and lipids in diabetic fruit fly which are induced by the high-sugar and high-fat diet
Jinyinhua, Sangbaipi, Zexie, Baizhu, Huzhang, and Xiakucao (13)	Anti-diabetic effects based on both growth rate and body size of fruit fly, as well as potential hypoglycemic effects
Danshen (76)	Regulating the glycolipid metabolism in the metabolic disorder model of fruit fly and returning it to normal effectively
Jinqi Jiangtang Tablets (62)	Reducing the level of glycolipid in fruit flies fed with high-fat diet significantly
The Clear Circulation Hot Party (61)	Decreasing the trehalose and triglyceride contents
Danqi Tablets (77)	Improving insulin resistance effectively

### Single medications

It was found that Yejuhua (*Chrysanthemum indicum* L.), Duhuo (*Angelica pubescens* Maxim.f. *biserrata* Shan et Yuan), Juemingzi (*Cassia obtusifolia* L.), Lizhihe (*Litchi chinensis* Sonn.), Baizhi [*Angelica dahurica* (Fisch.ex Hoffm.) Benth. et Hook.f.], Machixian (*Portulaca oleracea* L.), Yanhusuo (*Corydalis yanhusuo* W.T.Wang), Zhuling [*Polyporus umbellatus* (Pers.) Fries], Baiqian [*Cynanchum stauntonii* (Decne.) Schltr.ex Lévl.], Yinchen (*Artemisia capillaris* Thunb.), Rougui (*Cinnamomum cassia* Presl) (10) had alleviating effects on the growth and development of insulin mutant diabetic fruit fly, and Duhuo (*Angelica pubescens* Maxim.f. *biserrata* Shan et Yuan), Juemingzi (*Cassia obtusifolia* L.), Lizhihe (*Litchi chinensis* Sonn.) can improve lipid metabolism disorders and prolong lifespan. Aqueous extract of Bailian [*Ampelopsis japonica* (Thunb.) Makino] (73) relieves insulin resistance induced by a high-sugar diet *via* regulating the accumulation of lipids. At the same time, this study screens 15 herbs out of kinds of traditional Chinese medicines, including Pugongying (*Taraxacum mongolicum* Hand. -Mazz), Zihuadiding (*Viola yedoensis* Makino), Lugen (*Phragmites communis* Trin.), Digupi (*Lycium chinense* Mill.), Aiye (*Artemisia argyi* Levl. et Vant.), Zexie [*Alisma orientale* (Sam.) Juzep.], Yinyanghuo (*Epimedium brevicornu* Maxim.), Pipaye [*Eriobotrya japonica* (Thunb.) Lindl.], Juhe (*Citrus reticulata* Blanco), for their alleviating effects on insulin resistance induced by the high-sugar diet in fruit fly. Feeding with Gouqizi (*Lycium barbarum* L.) extract (74) can improve the situation, such as growth retardation and abnormal accumulation of lipid, which are caused by insulin resistance, in a dose-dependent manner. Shihu (*Dendrobium nobile* Lindl.) and Gegen (*Pueraria thomsonii* Benth.) (75) is synergistic to lower blood glucose and lipids in diabetic fruit flies which are induced by the high-sugar and high-fat diet. In addition, Jinyinhua (*Lonicera japonica* Thunb.), Sangbaipi (*Morus alba* L.), Zexie [*Alisma orientale* (Sam.) Juzep.], Baizhu (*Atractylodes macrocephala* Koidz.), Huzhang (*Polygonum cuspidatum* Sieb. et Zucc.), and Xiakucao (*Prunella vulgaris* L.) are suggested to have anti-diabetic effects based on both growth rate and body size of fruit fly, and they are also considered to have potential hypoglycemic effects (13).

Danshen’s (*Salvia miltiorrhiza* Bge.) stem and leaf extract (76) can effectively regulate the glycolipid metabolism in the metabolic disorder model of fruit flies and return it to normal. Through metabolomics analysis, it is found that the levels of stearic acid, cholesterol glucuronide, D-caprolactone, phosphatidic acid, deoxyribose, cysteine, kynurenine, 3-methylthiopropionate, glyceric acid, and gentianal are upregulated in the model group of fruit fly, and *N*-acetyl vitamins, cephalin, lecithin, and histidine are downregulated by Danshen (*Salvia miltiorrhiza* Bge.) treatment. Therefore, it can be concluded that the Danshen (*Salvia miltiorrhiza* Bge.) stem and leaf extract exert their effects on the regulation of glycolipid metabolism in fruit flies probably through metabolic pathways, including histidine metabolism, glycerophospholipid metabolism, pentose, and glucuronate interactions, and glyceride metabolism.

### Complex prescriptions

Jinqi Jiangtang Tablets (62) [composition: *Coptis chinensis* Franch., *Astragalus membranaceus* (Fisch.) Bge. var. *Mongholicus* (Bge.) Hsiao and *Lonicera japonica* Thunb.] could significantly reduce the level of glycolipid in fruit flies fed with a high-fat diet. The Clear Circulation Hot Party (61) [composition: *Euonymus alatus* (Thunb.) Sieb. and *Litchi chinensis* Sonn.] was studied in models of *Drosophila* diabetes which was induced by high-sugar and high fat, respectively and found that the trehalose and triglyceride contents were both decreased in HSD and HFD models. Danqi Tablets (77) [*Salvia miltiorrhiza* Bge. and *Panax notoginseng* (Burk.) F. H. Chen] was effective in improving insulin resistance which was caused by HSD and HFD by regulating glucose and lipid metabolism.

## Conclusion

At present, many traditional Chinese medicines have shown significant effects on improving the clinical symptoms of diabetic patients. The known anti-diabetic active ingredients of traditional Chinese medicines include polysaccharides, flavonoids, saponins, and alkaloids (77–79). The studies based on the *Drosophila* diabetes model have notably enhanced this standpoint. However, the components of traditional Chinese medicines are complex, and only a little number of bioactive compounds from them have been well-elucidated. Thus, traditional medicines may also have potential adverse effects besides potential benefits. The identification of the bioactive compounds, which can determine their physiological effect at the molecular level from such medicines or natural products, is of paramount importance in the future.

The *Drosophila* diabetes models supply a highly suitable system for discovering bioactive compounds or compounds combined with traditional Chinese medicines for DM management, improving the research of the underlying mechanisms at the molecular level, and thus diversifying our approaches against this disease. In Addition, insulin deficiency in Drosophila leads to growth retardation and reduced body length, which is reflected in both larvae and adults. As an easily observable phenotype, body length facilitates the discovery of small molecule compounds that promote the growth of insulin secretion-deficient mutant Drosophila through high-throughput screening. Its application in drug discovery, screening, and validation stages is promising. Although the molecular mechanisms of metabolic processes in Drosophila are to an extent similar to that in humans, the regulatory basis of metabolic processes also varies from humans and therefore drugs that are clinically effective may not exhibit effects in Drosophila. For example, sulphonylureas are effective in insulin-deficient Drosophila, whereas the effectiveness of metformin could not be confirmed by the above-mentioned phenotype in Drosophila.

## Author contributions

YM, RC, XW, JZ, WT, ZZ, and YL: writing—original draft. YM: writing—review and editing. QX: study conception and design, investigation, and supervision. All authors contributed to the article and approved the submitted version.
